# Evaluation of a ruminally protected blend of pantothenic acid, pyridoxine, folic acid, biotin, and vitamin B12 on finishing steer growth performance, efficiency of dietary net energy utilization, carcass trait responses, and liver abscess prevalence and severity

**DOI:** 10.1093/tas/txad084

**Published:** 2023-07-21

**Authors:** Thiago Lauro Maia Ribeiro, Forest L Francis, Jeff S Heldt, Warren C Rusche, Zachary K Smith

**Affiliations:** Department of Animal Science, South Dakota State University, Brookings, SD 57007, USA; Department of Animal Science, South Dakota State University, Brookings, SD 57007, USA; Selko USA, Indianapolis, IN 46241, USA; Department of Animal Science, South Dakota State University, Brookings, SD 57007, USA; Department of Animal Science, South Dakota State University, Brookings, SD 57007, USA

**Keywords:** beef, feedlot, finishing period, steer, protected vitamin B

## Abstract

The objective of this study was to determine the influence that a ruminally-protected B-vitamin (RPBV) blend (containing vitamin B5, B6, B7, B9, and B12) had on growth performance, efficiency of dietary net energy utilization, carcass trait responses, and liver abscess severity and prevalence in beef steers fed a finishing diet. Steers (*n* = 246; initial shrunk body weight [BW] = 411 ± 25.8 kg) from two different sources, were used in a 126-d RCBD experiment. Within 48 h after arrival, steers were individually weighed and allotted to 1 of 24 pens (*n* = 8 to 12 steers; 8 pens per treatment) and randomly assigned to 1 of 3 treatments: (1) No RPBV; (2) RPBV1 at 1 g/steer d^−1^; 3) RPBV2 at 2 g/steer d^−1^. During the first 14 d, cattle received two transition diets with increasing concentrate. From days 15 to 126, cattle were fed the final diet containing 53% dry-rolled corn; 23% corn silage; 20% MDGS; and 4% suspended supplement. On the first 28 d, steers of RPBV1 had a greater average daily gain (ADG) and better feed conversion (G:F), both by 9% (quadratic effect, *P* ≤ 0.02). However, cumulatively, no differences (*P* ≥ 0.13) among treatments were found for dry-matter intake (DMI), live final BW, ADG, or G:F. Carcass-adjusted final BW, ADG, and G:F were not influenced by treatment (*P* ≥ 0.59). Additionally, carcass weight, dressing percentage, marbling score, kidney–pelvic–heart fat, or BW at 28% empty body fat did not differ among treatments (*P* ≥ 0.11). Ribeye area (REA) was altered (quadratic effect, *P* = 0.02) by treatment; steers from RPBV1 had decreased REA compared to others. Additionally, calculated yield grade (YG) and calculated retail yield (RY) were altered (quadratic effect, *P* ≤ 0.01) by treatment; steers from RPBV1 had increased YG and decreased RY compared to others. Estimated empty body fatness tended (*P* = 0.06) to be greater from steers-fed RPBV compared to control. Overall USDA YG distribution was altered by dietary treatment (*P* = 0.01). The proportions of YG1 and YG5 carcasses were unaffected by treatment, but there was a shift in the proportion of carcasses that graded YG2, YG3, and YG4 among treatments. Distribution of USDA Quality Grade was not altered by treatment (*P* = 0.53). No treatment differences in liver abscess incidence or severity were observed (*P* = 0.13). The use of RPBV altered carcass muscularity and rib fat accumulation affecting the overall YG distribution. However, RPBV did not appreciably influence any cumulative growth performance measures or liver abscess outcome.

## Introduction

B-complex vitamins are water-soluble molecules that play a crucial role in various metabolic processes in animals, such as energy, carbohydrate, lipid, and amino acid metabolism. However, knowledge regarding B-vitamin requirements for feedlot beef cattle is limited. Since ruminal microorganisms synthesize B-complex vitamins during the fermentation of feed, it has been assumed that microbial production would be sufficient to meet the host’s requirements (Zinn et al., 1987; Morrison et al., 2018; Kaur et al., 2019). However, changes in the forage-to-concentrate ratio of the diet are known to alter the microbial activity of the rumen (Allison, 1965) and are therefore likely to affect the amount of vitamins produced.

Considering the remarkable advancements in genetic potential observed in modern ruminants, coupled with the intricate interplay of various factors that influence the synthesis of B vitamins, it has become increasingly important to address their nutritional needs adequately through dietary supplementation (Capper, 2011; Deters et al., 2021). While a limited number of studies have shown the potential benefits of incorporating B vitamins into the diet of beef cattle, the literature presents a diverse range of findings. Some researchers have reported notable improvements in ADG and overall growth performance upon administering B vitamin supplements (Deters et al., 2021; Evans, 2015). However, contrasting results have been observed, where others did not identify any significant enhancement in growth, efficiency of gain, or carcass outcomes (Clifford et al., 1967, Zinn et al., 1987, Word et al., 2022). Consequently, further investigation is imperative to ascertain the precise dietary requirements for B vitamins of modern ruminants to develop comprehensive strategies to optimize the health and productivity of ruminant livestock in a manner that aligns with evolving agricultural practices and animal welfare standards.

The objective of this study was to determine the influence that a rumen-protected B-vitamin blend (containing pantothenic acid, pyridoxine, folic acid, biotin, and cyanocobalamin; Vivalto—Selko USA, Indianapolis, IN) has on growth performance, efficiency of dietary net energy (NE) utilization, carcass trait responses, and liver abscess severity and prevalence in yearling beef steers that had grazed native summer pasture and fed a finisher based upon corn and corn coproducts.

## Methods and Materials

### Use of Animal Subjects

The animal care and handling protocols used in this study were approved by the South Dakota State University Institutional Animal Care and Use Committee #3958-01. This study was conducted at the Southeast Research Farm (SERF) of the South Dakota Agricultural Experiment Station located near Beresford, South Dakota, between 1 October 2021 and 2 February 2022.

### Animal Description and Initial Processing

Crossbred beef steers (*n* = 246) were sourced as two separate consignments from local South Dakota auction facilities approximately 5 d prior to the initiation of the experiment. Steers were allocated according to the source from pens 1 to 24. Approximately 48 h after arrival (day −3), steers were tagged, vaccinated against viral respiratory (Bovi-Shield Gold 5, Zoetis, Parsippany, NJ) and clostridia pathogens (Ultrabac 7/Somubac, Zoetis), and administered pour-on moxidectin (Cydectin, Bayer, Shawnee Mission, KS) according to label instructions. The body weight (BW) collected on day −3 was used for allotment purposes. Steers were selected for study enrollment based upon uniformity of BW and temperament on day −3. The steers were weighed on day 1 and subsequently allocated to their designated pens. The pens were 35 × 14 m open dirt lot with 6 m of linear bunk space and equipped with a heated, continuous flow waterer. The average of the BW measurements collected on day −3 and day 1 was used as the initial BW (*n* = 246 steers; initial average shrunk BW = 411 ± 25.4 kg). All steers were implanted on day 28 (98 d before harvest) with a 200 mg trenbolone acetate and 28 mg estradiol benzoate implant (Synovex Plus, Zoetis, Parsippany, NJ). All implants were checked on day 56 (28 d following implantation) and any implant abnormalities were noted, steers identified with missing, or abscessed-out implants were administered to another implant. All steers were fed ractopamine HCl at a rate of 300 mg per steer daily for the final 28 d prior to harvest.

### Experimental Design and Treatments

Pens were assigned to 1 of 3 treatments with 8 replicate pens (24 pens total) assigned to each treatment in a RCBD. Treatments included (1) fed no rumen-protected B-vitamin blend (RPBV0) 2) fed the rumen-protected B-vitamin blend (Vivalto—Selko USA, Indianapolis IN) at a rate of 1.0 g/steer·d^−1^ and provided (mg/steer daily) 36 mg of pantothenic acid, 22.6 mg of pyridoxine, 3.6 mg of folic acid, 2.9 mg of biotin, and 0.4 mg of vitamin B12 (RPBV1); 3) fed the rumen-protected B-vitamin blend at a rate of 2.0 g/steer·d^−1^ and provided (mg per steer daily) 72.0 mg of pantothenic acid, 45.2 mg of pyridoxine, 7.2 mg of folic acid, 5.8 mg of biotin, and 0.8 mg of vitamin B12 (RPBV2). The B vitamins were protected by a proprietary dried fat spray resulting in a 95% ruminal passage rate of the B vitamins. The dietary treatment was included in a ground corn carrier and offered in replacement of dry-rolled corn at a rate of 45.4 g/steer·d^−1^ (as-fed basis).

### Dietary Management

Cattle were fed once a day at 0730 h and bunks were managed for ad libitum access (slick bunk management approach) to feed with minimal day-to-day variation in the amount of feed not consumed being the primary target for feed calls. Cattle were transitioned to the 90% concentrate diet, over the course of 14 d (Table 1). The final diet (Table 2) was fed from day 15 until day 126 (days before harvest).

**Table 1.** Actual ingredient inclusion and nutrient content based upon feed batching records

Item	Step 1 (days 1 to 7)	Step 2 (days 8 to 14)
Dry-rolled corn	35.13	46.24
Modified distillers grains plus solubles	17.83	17.92
Corn silage	24.00	21.89
Grass Hay	19.20	10.01
Suspended supplement^*^	3.84	3.94
Analyzed nutrient composition		
DM^†^, % as fed	59.18	54.65
CP^‡^, %	13.36	13.49
ADF^§^, %	18.87	14.15
NDF^¶^, %	31.34	24.61
Ash, %	6.91	6.07
EE^**^, %	3.61	3.70
NEg^††^, Mcal/kg	1.19	1.28

^*^Contained on a DM basis: 30, 844 IU of Vitamin A/kg, 170 IU of Vitamin E/kg, 827 g of Monensin/ metric ton (Elanco Animal Health, Greenfield, IN), and 165 g of Tylosin/metric ton (Elanco Animal Health).

^†^Dry matter.

^‡^Crude protein.

^§^Acid detergent fiber.

^¶^Neutral detergent fiber.

^**^Ether extract.

^††^Net energy for gain.

**Table 2.** Composition of finishing diet (dry-matter basis)^*^

Ingredient	Finishing diet fed from d 15 to 126
Dry-rolled corn	53.18
Modified distillers grains plus solubles	19.93
Corn silage	23.10
Suspended supplement^†^	3.79
Analyzed nutrient composition	
DM^‡^^,^^¶¶^, % as fed	59.97
CP^§^^,^^¶¶^, %	13.41
NPN^¶^, %	1.35
ADF^**^^,^^¶¶^, %	10.11
NDF^††^^,^^¶¶^, %	20.85
Ash^¶¶^, %	5.17
EE^‡‡^^,^^¶¶^, %	3.91
NEg^§§^^,^¶¶^,^, Mcal/kg	1.37
Calcium, %	0.66
Magnesium, %	0.25
Phosphorus, %	0.46
Potassium, %	0.96
Sulfur, %	0.20
Cobalt, mg/kg	0.50
Copper, ppm	12.00
Iron, ppm	109
Manganese, ppm	40.00
Selenium, ppm	0.5
Zinc, ppm	106

^*^All steers received 300 mg per steer daily ractopamine hydrochloride for the final 28 d on feed.

^†^Contained on a dry-matter basis: 30, 844 IU of Vitamin A/kg, 170 IU of Vitamin E/kg, 827 g of monensin sodium/ metric ton (Elanco Animal Health, Greenfield, IN) 165 g of tylosin phosphate/metric ton (Elanco Animal Health), 250.00 ppm copper, 500.00 ppm manganese, and 2,250.00 ppm zinc,.

^‡^Dry matter.

^§^Crude protein.

^¶^Nonprotein nitrogen, CP equivalent.

^**^Acid detergent fiber.

^††^Neutral detergent fiber.

^‡‡^Ether extract.

^§§^Net energy for gain.

^¶¶^Weekly ingredient samples were stored in a freezer at −20 °C until nutrient analyses were completed. After weekly DM determination (method no. 935.29), weekly samples from each ingredient were composited by month and analyzed for N (method no. 968.06; Rapid Max N Exceed; Elementar; Mt. Laurel, NJ), and ash (method no. 942.05) content (AOAC, 2012, 2016). Modified distillers grains plus soluble samples were analyzed for ether extract content using an Ankom Fat Extractor (XT10; Ankom Technology, Macedon, NY). Percentages of ADF and NDF were assumed to be 3% and 9% for dry-rolled corn (Preston, 2016). Analysis of ADF and NDF composition for all other feeds was conducted as described by Goering and Van Soest et al. (1970). Diets presented her are actual DM diet composition, actual nutrient concentrations, and tabular energy values (Preston, 2016).

The liquid supplement was included at 4% of the diet and provided to the diet (DM basis) 827 g of Monensin/metric ton (Elanco Animal Health, Greenfield, IN), and 165 g of Tylosin/metric ton (Elanco Animal Health) and the inorganic trace minerals were added to the supplement to meet the nutrient requirements for growing beef steers (NASEM, 2016).

Feed was manufactured in a mixer (4.16 m^3^; 8 pens fed per batch) that was mounted on scales and connected to a tractor, all ingredients were added into the mixer to the nearest 0.9 kg, and feed was delivered to each pen separately (weighed out of the mixer to the nearest 0.9 kg). Batching sequence was RPBV0 (8 pens), RPBV1 (8 pens), and RPBV2 (8 pens). Following each batch of feed, long-stem grass hay (~4.54 kg) was added to the mixer and used to flush out all residual feed remaining in the mixer. Mixing of the following batch did not occur until the scale head read 0 to 1 kg.

Feedstuff samples were taken weekly and analyzed for DM content. Weekly ingredient samples were composited monthly for CP, NDF, ADF, and ash determination using AOAC procedures. A single TMR sample was collected and analyzed for Ca, P, Co, Cu, Mn, Mo, Se, and Zn according to AOAC procedures. Orts were collected, weighed, and dried in a forced air oven at 100 °C for 24 h in order to determine DM content. The DMI of each pen was adjusted to reflect the total DM delivered to each pen after subtracting the quantity of dry orts for each interim period.

Weekly ingredient samples were stored in a freezer at −20 °C until nutrient analyses were completed. After weekly DM determination (method no. 935.29), weekly samples from each ingredient were composited by month and analyzed for N (method no. 968.06; Rapid Max N Exceed; Elementar; Mt. Laurel, NJ) and ash (method no. 942.05) content (AOAC, 2012, 2016). Modified distiller grains plus soluble samples were analyzed for ether extract content using an Ankom Fat Extractor (XT10; Ankom Technology, Macedon, NY). Percentages of ADF and NDF were assumed to be 3% and 9% for dry-rolled corn (Preston, 2016). Analysis of ADF and NDF composition for all other feeds was conducted as described by Goering and VanSoest (1970). The diets presented in Tables 1 and 2 are actual DM diet composition, actual nutrient concentrations, and tabular energy values (Preston, 2016).

### Growth Performance Calculations

Steers were individually weighed on days −3, 1, 28, 56, 98 (start RH), and 126 (days before harvest). Cumulative growth performance was calculated on live and carcass-adjusted basis. Initial BW was the average of the day −3 and day 1 BW. All live BW measures used were pencil shrunk by 4% to account for gastrointestinal tract fill and carcass-adjusted final BW was calculated from hot carcass weight (HCW) divided by 0.625. Average daily gain was determined as the difference between final and initial BW divided by days on feed (126 d). DMI was tabulated at weekly intervals and summarized by interim period. Feed conversion ratio was calculated using ADG divided by DMI.

Growth performance (live basis) was used to calculate performance-based dietary NE in order to determine the efficiency of dietary NE utilization. The performance-based dietary NE was calculated from daily energy gain (EG; Mcal/d): EG = ADG^1.097^ × 0.0557*W*^0.75^, where *W* is the mean equivalent shrunk BW (kg; NASEM, 1996) from median feeding shrunk BW and final BW at 28% estimated empty body fatness (AFBW) calculated as: [median feeding shrunk BW × (478/AFBW), kg; (NRC, 1996)]. Maintenance energy (EM) was calculated by the equation: EM = 0.077 × median feeding shrunk BW^0.75^. DMI is related to energy requirements and dietary NEm (Mcal/kg) according to the following equation: DMI = EG/(0.877NEm − 0.41), and can be resolved for estimation of dietary NEm by means of the quadratic formula *x* = (−*b*±√[*b*^2^ − 4*ac*])/2*c*, where *a* = −0.41EM, *b* = 0.877EM + 0.41DMI + EG, and *c* = −0.877DMI (Zinn and Shen, 1998). Dietary NEg was derived from NEm using the following equation: NEg = 0.877NEm − 0.41 (Zinn, 1987).

Steers were marketed and harvested at a commercial abattoir when steers were estimated by visual appraisal to have 1.40 cm of rib fat at the 12th rib. Steers were loaded onto trucks, shipped 80 km, and harvested the following morning. Liver abscess prevalence and severity were determined by a trained technician using the Elanco system as Normal (no abscesses), A− (1 or 2 small abscesses or abscess scars), A (2 to 4 well-organized abscesses less than 2.54 cm. diameter), or A+ (one or more large active abscesses greater than 2.54 cm diameter with inflammation of surrounding tissue). Video image data were obtained from the plant for rib eye area, rib fat, kidney–pelvic–heart fat, calculated USDA Yield Grade, and USDA marbling scores. Dressing percentage was calculated as HCW/(final BW × 0.96). Estimated empty body fat (EBF) percentage and AFBW were calculated from observed carcass traits (Guiroy et al., 2002), and the proportion of closely trimmed boneless retail cuts from carcass round, loin, rib, and chuck was determined according to the equation described by Murphey et al. (1960).

### Management of Pulls and Removals

A total of five steers were removed from the experiment for reasons not related to dietary treatment. One steer was removed due to hardware disease (RPBV1), one steer was removed due to musculoskeletal issues (RPBV1), one steer was removed for overall poor gain (RPBV2), and two steers were found dead due to a perforated reticulo-rumen (both from RPBV2).

### Statistical Analysis

Growth performance, carcass traits, and efficiency of dietary energy utilization were analyzed as an RCBD using the MIXED procedure of SAS 9.4 (SAS Inst. Inc., Cary, NC) with pen as the experimental unit. The model included the fixed effect of dietary treatment and block was considered a random variable. Distribution of USDA Yield and Quality grade, as well as liver abscess severity and prevalence data, was analyzed as multinomial distributions using the GLIMMIX procedure of SAS 9.4 to identify differences in the distributions among treatments. Individual steer served as the experimental unit for categorical outcome data and the same fixed and random effects in the model as described previously were used. The model specified a solution function for the multinomial response, with the number of animals slaughtered identified in the denominator. Least squares means were generated using the LSMEANS statement of SAS 9.4. Treatment effects were evaluated by the use of orthogonal polynomials (Steel and Torrie, 1960). An *a* of 0.05 was used to determine significance, and an *a* of 0.06 to 0.10 was considered a tendency.

## Results and Discussion

The majority of studies investigating the effects of B-vitamin supplementation have been with dairy cattle, with findings indicating enhancements in neutrophil function and improvements in overall immune response. Furthermore, more efficient glucose metabolism has also been observed in these studies (Pinotti et al., 2005; Ghorbani et al., 2008), reduced heat stress (Wrinkle et al., 2012), improvements in the synthesis of keratin, and reduced sole ulcers (Bergsten et al., 2003). Additionally, B-vitamin supplementation has been associated with increased total milk, protein, and fat yield in dairy cows (Graulet et al., 2007; Sacadura et al., 2008; Karkoodi and Tamizrad, 2009; Duplessis et al., 2014). However, in contrast to these positive responses, the current study did not observe any performance benefits when supplementing an RPBV to yearling feedlot steers.

From days 1 to 28, steers of RPBV1 had a 9% improvement in ADG and feed efficiency (quadratic effect; *P* ≤ 0.02) compared to the other treatments (Table 3). However, DMI was not appreciably influenced by the addition of a rumen-protected B-vitamin blend (*P* ≥ 0.05). Leclerc et al. (2015) conducted a study to assess the impact of feeding 2 g/steer·d^−1^ of rumen-protected B vitamins (folic acid, pyridoxine, pantothenic acid, and biotin) on the performance of beef cattle during a 21-d receiving period. The study yielded similar results to those observed during this trial. Specifically, the ADG and G:F were significantly improved (*P* < 0.05), while DMI remained unaffected. Furthermore, Evans et al. (2015) fed steers with 2 g/steer·d^−1^ of rumen-protected B vitamins (folic acid, pyridoxine, pantothenic acid, and biotin) during a 21-d receiving period. They observed an increase in final BW, ADG, and G:F, without any significant change in DMI. Certain B vitamins play vital roles in energy and protein metabolism (Manore, 2000). These essential nutrients become especially crucial during periods of stress, such as weaning, shipping, and the introduction to new environments (Evans et al., 2015). These stressors increase the metabolic demands of the animal, leading to an increased requirement for B vitamins. Additionally, the stress-induced metabolic shifts and decreased ruminal activity further contribute to the elevated needs for these essential nutrients (Evans et al., 2015; Morrison et al., 2018). From days 29 to 56, the use of RPBV did not appreciably influence BW, DMI, or ADG, but tended to influence (quadratic effect; *P *= 0.10) feed efficiency (Table 3).

**Table 3.** Finishing steer growth performance and efficiency of dietary net energy utilization

Item	RPBV^*^, g/hd·d^−1^				*P*-value	
	0	1	2	SEM	Linear	Quadratic
Pens, *n*	8	8	8	—	—	—
Steers, *n*	82	80	79	—	—	—
Initial BW^†^, kg	412	411	412	—	—	—
Initial to day 28						
d 28 BW, kg	457	460	458	4.7	0.69	0.14
ADG^‡^, kg	1.60	1.76	1.61	0.143	0.88	0.02
DMI^§^, kg	8.71	8.79	8.82	0.177	0.20	0.73
G:F^¶^	0.183	0.200	0.182	0.0066	0.89	0.01
F:G	5.46	5.01	5.49	—	—	—
Days 29 to 56						
Day 56 BW, kg	532	533	536	7.4	0.28	0.82
ADG^‡^, kg	2.69	2.62	2.80	0.261	0.40	0.23
DMI^§^, kg	12.22	12.53	12.52	0.444	0.15	0.38
G:F^¶^	0.221	0.209	0.223	0.0087	0.72	0.10
F:G	4.53	4.79	4.47	—	—	—
Live-weight basis						
Final BW^†^, kg	651	655	655	4.2	0.43	0.54
ADG^‡^, kg	1.90	1.94	1.92	0.034	0.52	0.34
DMI^§^, kg	12.01	12.27	12.21	0.168	0.25	0.27
G:F^¶^	0.158	0.158	0.158	0.0022	0.72	0.94
Carcass-adjusted basis^**^						
Final BW, kg	675	677	677	6.2	0.68	0.84
ADG, kg	2.09	2.11	2.10	0.044	0.75	0.65
DMI, kg	12.01	12.27	12.21	0.168	0.25	0.27
G:F	0.174	0.172	0.172	0.0030	0.64	0.79
Performance-adjusted dietary net energy (NE)^††^, Mcal/kg						
Maintenance	2.00	2.00	1.99	0.018	0.79	0.83
Gain	1.35	1.35	1.34	0.016	0.79	0.83
Observed-to-expected NE						
Maintenance	0.99	0.99	0.99	0.009	0.79	0.83
Gain	0.99	0.99	0.99	0.012	0.79	0.83

^*^Treatments included (1) fed no rumen-protected B-vitamin blend (RPBV0); (2) fed the rumen-protected B-vitamin blend at a rate of 1.0 g/steer·d^−1^ and provided (mg/steer daily) 36 mg of pantothenic acid, 22.6 mg of pyridoxine, 3.6 mg of folic acid, 2.9 mg of biotin, and 0.4 mg of vitamin B12 (RPBV1); (3) fed the rumen-protected B-vitamin blend at a rate of 2.0 g/steer·d^−1^ and provided (mg per steer daily) 72.0 mg of pantothenic acid, 45.2 mg of pyridoxine, 7.2 mg of folic acid, 5.8 mg of biotin, and 0.8 mg of vitamin B12 (RPBV2).

^†^Body weight (BW) was pencil shrunk 4% to account for digestive tract fill.

^‡^ADG = average daily gain

^§^DMI = dry-matter intake

^¶^G:F = gain-to-feed ratio (ADG/DMI)

^**^Calculated as: Final BW = HCW/0.625

^††^Calculated from body weights pencil shrunk 4%.

Cumulative growth performance responses are shown in Table 3. DMI was not appreciably influenced by the addition of RPBV (*P* ≥ 0.13). Live-basis final BW, ADG, and G:F were not different among treatments (*P* ≥ 0.25), agreeing with results in previous studies (Clifford et al., 1967, Zinn et al., 1987, Word et al., 2022). However, Deters et al. (2021) fed different levels of rumen-protected folic acid (0, 30, 60, 90, 120, or 150 mg/steer d^−1^) and reported improved ADG and G:F during the growing period, with linear increases in DMI. Carcass-adjusted final BW, ADG, nor G:F was not appreciably influenced by dietary treatment (*P* ≥ 0.59). Word et al. (2022) fed 3.0 mg/kg DM of rumen-protected folic acid observed that carcass-adjusted performance and HCW were numerically improved when supplementing a novel source of rumen-protected folate and Co; however, live growth performance was not affected by treatment. Observed dietary NE values for maintenance and gain based upon growth performance were not influenced by the addition of RPBV in the current experiment. The ratio of observed-to-expected dietary NE values was in close agreement with expectations (0.99) and was not influenced by dietary treatment (*P* ≥ 0.79). Hence growth performance was consistent with measures of DMI and tabular energy value of the diet.

Carcass trait responses are located in Table 4. HCW, dressing percentage, marbling, kidney–pelvic–heart fat, or BW at 28% EBF (AFBW) did not differ due to dietary treatment (*P* ≥ 0.11). Limited research exists on the specific impact of B-vitamin supplementation on carcass characteristics in cattle. However, Word et al. (2022) observed that dressing percentage was improved on steers-fed RPBV compared to the control with no differences in REA, marbling, or EBF. In the current experiment, REA was altered by treatment (quadratic effect, *P* = 0.02), with steers from RPBV1 having decreased REA compared to others. Hence, calculated yield grade (YG) and percentage of wholesale cuts obtained from the round, loin, rib, and chuck (retail yield) were also altered by dietary treatment (quadratic effect, *P* ≤ 0.01) with steers from RPBV1 having increased YG and decreased retail yield compared to steers-fed RBPV0 or RPBV2. Estimated empty body fatness tended to be greater from steers-fed RPBV compared to control (*P* = 0.06) presumably caused by numerically greater RF depth and lesser REA.

**Table 4.** Carcass traits of finishing beef steers

Item	RPBV^*^, g/hd·d^−1^			SEM	*P*-value	
	0	1	2		Linear	Quadratic
Pens, *n*	8	8	8	-	-	-
Steers, *n*	82	80	79	-	-	-
Hot carcass weight, kg	422	423	424	3.9	0.68	0.84
Dressing percentage^†^, %	64.74	64.58	64.66	0.342	0.82	0.70
Rib fat, cm	1.32	1.42	1.40	0.051	0.28	0.15
Ribeye area, cm^2^	90.2	87.0	89.3	0.929	0.47	0.02
Marbling^‡^	509	508	516	16.7	0.70	0.76
Kidney–pelvic–heart fat, %	1.79	1.81	1.81	0.029	0.51	0.81
Calculated yield grade	3.22	3.50	3.35	0.087	0.19	0.01
Retail Yield^§^, %	50.04	49.46	49.78	0.187	0.18	0.01
EBF^¶^, %	31.08	31.77	31.50	0.306	0.19	0.09
AFBW^**^, kg	610	601	606	6.6	0.53	0.27

^*^Treatments included (1) fed no rumen-protected B-vitamin blend (RPBV0); (2) fed the rumen-protected B-vitamin blend at a rate of 1.0 g/steer·d^−1^ and provided (mg per steer daily) 36 mg of pantothenic acid, 22.6 mg of pyridoxine, 3.6 mg of folic acid, 2.9 mg of biotin, and 0.4 mg of vitamin B12 (RPBV1); (3) fed the rumen-protected B-vitamin blend at a rate of 2.0 g/steer·d^−1^ and provided (mg/steer daily) 72.0 mg of pantothenic acid, 45.2 mg of pyridoxine, 7.2 mg of folic acid, 5.8 mg of biotin, and 0.8 mg of vitamin B12 (RPBV2).

^†^Dressing percentage was calculated as hot carcass weight/(final BW × 0.96).

^‡^400 = small^00^

^§^Proportion of closely trimmed boneless retail cuts from carcass round, loin, rib, and chuck were determined according to the equation described by Murphey et al. (1960).

^¶^Estimated empty body fat (EBF) percentage from observed carcass traits (Guiroy et al., 2002).

^**^Final shrunk body weight adjusted to 28% EBF (AFBW) according to Guiroy et al. (2002).

Categorical carcass outcomes are located in Table 5. The overall USDA Yield Grade distribution was altered by dietary treatment (*P* = 0.01). There were similar levels of YG1 and YG5 carcasses among treatments, but a shift in the distribution of YG with greater proportions of YG3 and 4 carcasses from supplemented steers and lesser YG2 compared to control. B vitamins are involved in lipid metabolism and energy production. Theoretically, supplemental B vitamins may influence the deposition and distribution of fat within the carcass, potentially resulting in YG changes by alterations in fat metabolism. Further studies are needed to investigate the direct impact of B vitamin supplementation on YG and carcass characteristics in yearling beef steers. Dietary treatment did not alter the overall distribution of USDA Quality Grade (*P* = 0.53), similar to results reported by Word et al. (2022).

**Table 5.** Effects of RPBV on overall distribution of yield and quality grade measurements and liver scores.

Item	RPBV^*^, g/hd·d^−1^			*P*-value
	0	1	2	*F*-test
Pens, *n*	8	8	8	—
Steers, *n*	82	80	79	—
Yield grade, %				
1	1.2	1.3	1.3	0.01
2	35.4	15.2	25.36	
3	50.0	60.8	59.5	
4	13.4	21.5	12.6	
5	0.0	1.3	1.3	
Quality grade, %				
Select	11.0	14.29	9.0	0.53
Choice	35.4	35.1	38.5	
Average choice	41.5	35.1	35.9	
Upper choice	8.5	11.7	15.4	
Prime	3.7	3.9	1.3	
Liver scores^†^, %				0.13
Normal	89.0	89.9	79.8	
A−	7.3	5.1	12.7	
A	0.0	0.0	1.3	
A+	3.7	5.1	6.3	

^*^Treatments included (1) fed no rumen-protected B-vitamin blend (RPBV0); (2) fed the rumen-protected B-vitamin blend at a rate of 1.0 g/steer·d^−1^ and provided (mg/steer daily) 36 mg of pantothenic acid, 22.6 mg of pyridoxine, 3.6 mg of folic acid, 2.9 mg of biotin, and 0.4 mg of vitamin B12 (RPBV1); (3) fed the rumen-protected B-vitamin blend at a rate of 2.0 g/steer·d^−1^ and provided (mg/steer daily) 72.0 mg of pantothenic acid, 45.2 mg of pyridoxine, 7.2 mg of folic acid, 5.8 mg of biotin, and 0.8 mg of vitamin B12 (RPBV2).

^†^Liver abscess prevalence and severity were determined by a trained technician using the Elanco system as Normal (no abscesses), A− (one or two small abscesses or abscess scars), A (2 to 4 well-organized abscesses less than 2.54 cm diameter), or A+ (one or more large active abscesses greater than 2.54 cm diameter with inflammation of surrounding tissue).

The overall distribution of liver scores was not altered by dietary treatment (*P* = 0.13). Steers from RPBV0 and RPBV1 had numerically more livers classified as normal compared to RPBV2 steers. Deters et al. (2021) observed that folic acid supplementation at 30 or 60 mg/kg reduced the incidence of abscessed livers. However, it was observed by Word et al.’s (2022) trial that the percentage of livers with abscesses tended to be reduced by B-vitamin supplementation (*P* = 0.12).

## Conclusion

The use of RPBV had no appreciable influence on any cumulative growth performance responses when fed to finishing steers in this experiment. When RPBV is used in finishing diets, carcass muscularity and rib fat accumulation were affected that in turn altered overall YG distribution. Additionally, RPBV application does not appear to reduce undesirable liver health outcomes.

## Data Availability

Data can be made available with reasonable request to Z.K.S.
